# Genetic Evidence of Middle East Respiratory Syndrome Coronavirus (MERS-Cov) and Widespread Seroprevalence among Camels in Kenya

**DOI:** 10.1007/s12250-018-0076-4

**Published:** 2018-12-20

**Authors:** Sheila Ommeh, Wei Zhang, Ali Zohaib, Jing Chen, Huajun Zhang, Ben Hu, Xing-Yi Ge, Xing-Lou Yang, Moses Masika, Vincent Obanda, Yun Luo, Shan Li, Cecilia Waruhiu, Bei Li, Yan Zhu, Desterio Ouma, Vincent Odendo, Lin-Fa Wang, Danielle E. Anderson, Jacqueline Lichoti, Erick Mungube, Francis Gakuya, Peng Zhou, Kisa-Juma Ngeiywa, Bing Yan, Bernard Agwanda, Zheng-Li Shi

**Affiliations:** 1 Institute for Biotechnology Research, Jomo Kenyatta University of Agriculture and Technology, Nairobi, 62000-00200 Kenya; 2 CAS Key Laboratory for Special Pathogens and Biosafety, Wuhan Institute of Virology, Chinese Academy of Sciences, Wuhan, 430071 China; 3 College of Biology, Hunan University, Changsha, 410006 China; 4 Department of Medical Microbiology, University of Nairobi, Nairobi, 30197-00100 Kenya; 5 Veterinary Services Department, Kenya Wildlife Service, Nairobi, 40241-00100 Kenya; 6 Veterinary Research Institute, Kenya Agriculture and Livestock Research Organization, Nairobi, 57811-00200 Kenya; 7 Veterinary Services Programme in Emerging Infectious Diseases, Duke-NUS Medical School, Singapore, 169857 Singapore; 8 Directorate of Veterinary Services, State Department of Livestock, Ministry of Agriculture, Livestock Fisheries and Irrigation, Nairobi, 34188-00100 Kenya; 9 Kenya Camel Association, Nairobi, 30095-00100 Kenya; 10 Department of Zoology, National Museums of Kenya, Nairobi, 40658-00100 Kenya

**Keywords:** Middle East respiratory syndrome coronavirus (MERS-CoV), One-health, Public health, Zoonosis, Kenya

## Abstract

We describe the first genome isolation of Middle East respiratory syndrome coronavirus (MERS-CoV) in Kenya. This fatal zoonotic pathogen was first described in the Kingdom of Saudi Arabia in 2012. Epidemiological and molecular evidence revealed zoonotic transmission from camels to humans and between humans. Currently, MERS-CoV is classified by the WHO as having high pandemic potential requiring greater surveillance. Previous studies of MERS-CoV in Kenya mainly focused on site-specific and archived camel and human serum samples for antibodies. We conducted active nationwide cross-sectional surveillance of camels and humans in Kenya, targeting both nasal swabs and plasma samples from 1,163 camels and 486 humans collected from January 2016 to June 2018. A total of 792 camel plasma samples were positive by ELISA. Seroprevalence increased with age, and the highest prevalence was observed in adult camels (82.37%, 95% confidence interval (CI) 79.50–84.91). More female camels were significantly seropositive (74.28%, 95% CI 71.14–77.19) than male camels (*P* < 0.001) (53.74%, 95% CI 48.48–58.90). Only 11 camel nasal swabs were positive for MERS-CoV by reverse transcription-quantitative PCR. Phylogenetic analysis of whole genome sequences showed that Kenyan MERS-CoV clustered within sub-clade C2, which is associated with the African clade, but did not contain signature deletions of *orf4b* in African viruses. None of the human plasma screened contained neutralizing antibodies against MERS-CoV. This study confirms the geographically widespread occurrence of MERS-CoV in Kenyan camels. Further one-health surveillance approaches in camels, wildlife, and human populations are needed.

**Electronic supplementary material:**

The online version of this article (10.1007/s12250-018-0076-4) contains supplementary material, which is available to authorized users.

## Introduction

Middle East respiratory syndrome coronavirus (MERS-CoV) is a positive sense, single-stranded RNA virus in the genus *Betacoronavirus*. It is a zoonotic pathogen capable of causing severe respiratory disease in humans. Dromedary camels are considered as a source of zoonotic origin and natural reservoir host for MERS-CoV (Azhar *et al.*^2014^; Haagmans *et al.*^2014^). As of September 20, 2018, MERS-CoV infection has been reported from 27 countries with 2249 laboratory-confirmed cases in humans and at least 798 related deaths (WHO 2018). Most of these cases occurred in the Kingdom of Saudi Arabia, where the high prevalence of MERS-CoV in dromedary camels and direct contact with infected camels have been linked to human infections (Haagmans *et al.*^2014^; Azhar *et al.*^2014^). Notably, most human cases outside of the Arabian Peninsula have been linked to travel from Saudi Arabia. Numerous surveillance studies in Africa have revealed the presence of MERS-CoV antibodies in dromedaries from several African countries including Nigeria, Egypt, and Mali (Chu *et al.*^2014^; Chu *et al.*^2018^; Chu *et al.*^2015^; Falzarano *et al.*^2017^), where camels are reared and people frequently travel for pilgrimage to Saudi Arabia.

Similarly, in Kenya, previous studies reported a high seroprevalence of MERS-CoV antibodies in archived dromedaries samples collected in 1992–2013 (Corman *et al.*^2014^), and serological infection of MERS-CoV was confirmed in two people sampled from Tana River County in 2013–2014 (Liljander *et al.*^2016^). However, a recent study reported no evidence of MERS-CoV infection among camel farmers (Munyua *et al.*^2017^). Other recent studies suggested that the high densities of camel populations may be correlated with increased seropositivity and may contribute to long-term maintenance of the virus in the camel population. In this study, we evaluated the exposure of camels and humans to MERS-CoV in all camel-rearing counties in Kenya as part of a country-wide surveillance program.

## Materials and Methods

### Study Area and Design

**Fig. 1:**
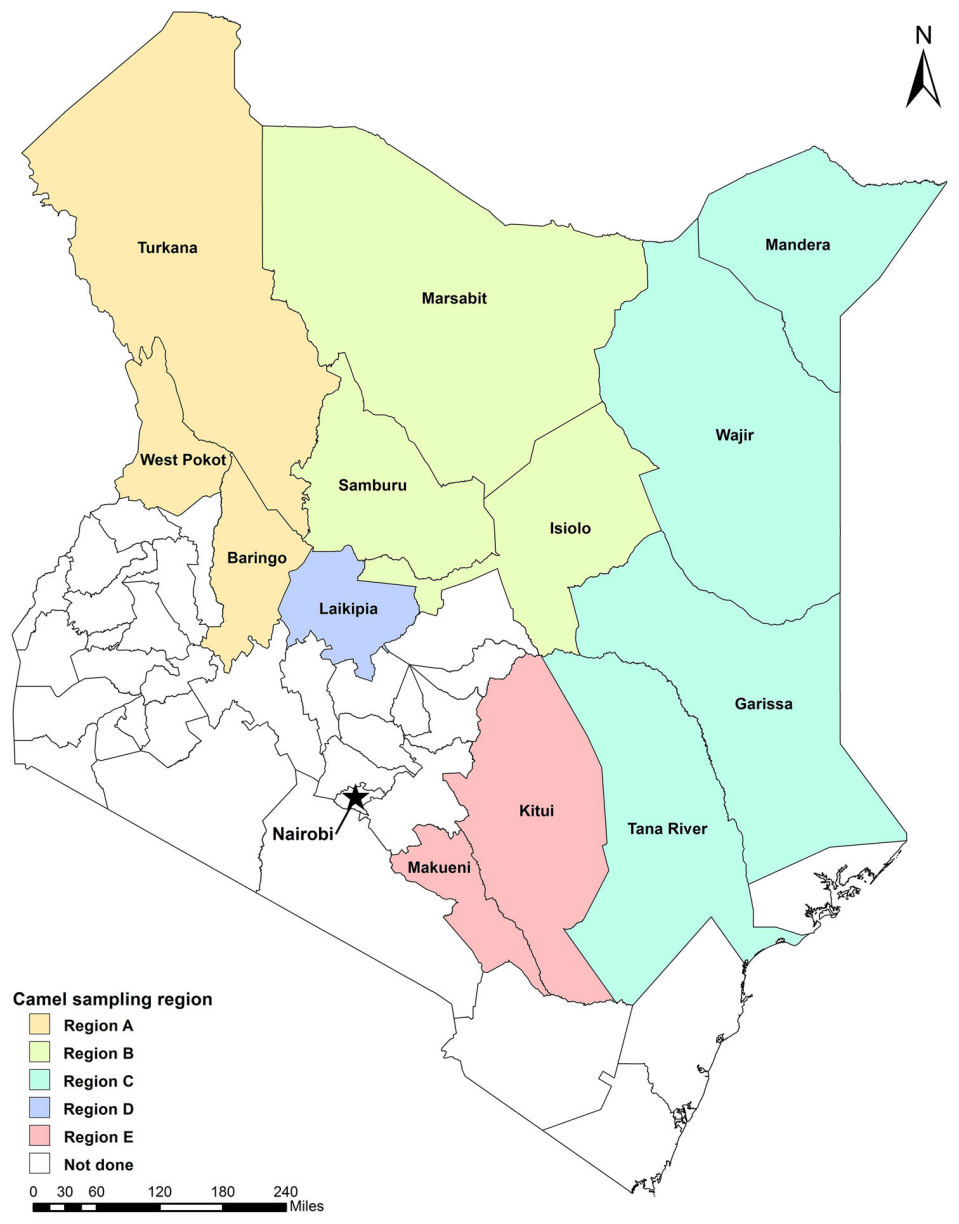
Map of Kenya showing the 13 counties surveyed in this study.

This cross-sectional study was carried out from January 2016 to June 2018 across 13 counties in Kenya where camels are reared (Table 1 and Fig. 1). The four camel breed types in Kenya sampled in this study were from historical and recent camel rearing tribes (Mburu *et al.*^2003^). Therefore, based on the geographical division of breed types, the 13 counties were grouped into five groups. For human sampling, high-risk groups such as camel herders and their immediate families were targeted. Sampling mainly focused on high-risk locations such as around watering points and common browsing locations that attract several herds of camels from different regions.

**Table 1:** Univariate analysis of factors associated with ELISA positive camels for MERS-CoV in Kenya.

Variable	Category	Ecotype/Breed	No. tested	No. positive	Prevalence % (95% CI)	Odds Ratio (95% CI)	*P* value
Region	Region A	Turkana	156	76	48.72 (95% CI 41.00–56.50)	4.72 (2.39–9.86)	*P *< 0.001
	Region B	Rendille/Gabbra	293	234	79.86 (95% CI 74.90–84.06)	19.62 (10.11–40.44)	
	Region C	Somali	611	460	75.29 (95% CI 71.72–78.54)	15.16 (8.17–30.01)	
	Region D	Improved/Pakistani	84	14	16.67% (95% CI 10.20–26.05)	1	
	Region E	Somali	19	8	42.11% (95% CI 23.14–63.72)	3.58 (1.05–12.00)	
Sex*	Male		348	187	53.74% (95% CI 48.48–58.90)	1	*P *< 0.001
	Female		801	595	74.28% (95% CI 71.14–77.19)	2.49 (1.89–3.26)	
Age*	Juvenile		319	115	36.05% (95% CI 30.98–41.46)	1	*P *< 0.001
	Sub-adult		70	41	58.57% (95% CI 46.88–69.37)	2.5 (1.43–4.42)	
	Adult		760	626	82.37% (95% CI 79.50–84.91)	8.27 (6.1–11.25)	

*Data for 14 samples were not available.

### Sampling Procedure

For camel sampling, camel owners were informed about the study in a language that they understood. The camels were physically restrained, and 10 mL of blood from the jugular vein was drawn into anti-coagulant vacuum blood collection tubes. Additionally, 1163 nasal swabs samples were collected in RNAlater^®^ (Ambion, Foster City, CA, USA) and virus transport medium.

The research was conducted in accordance with the Helsinki Declaration for sampling of human subjects. Informed consent was obtained from camel owners, handlers, and family members or their guardians (in case of underage children) from whom blood was collected. Five milliliters of blood from consenting study participants were collected by a clinician or phlebotomist from a peripheral vein into an anti-coagulant vacuum blood collection tube. Sociodemographic data were also collected from the participants (Supplemental Table S2). Other data such as age and sex were also collected.

Blood samples were centrifuged, plasma was aliquoted and stored in liquid nitrogen while in the field, and the samples were transported to Nairobi for storage at − 80 °C. A total of 1,163 dromedary camels were sampled from 13 counties and 486 human subjects from 10 counties. Camel and human samples were exported to Wuhan Institute of Virology, Chinese Academy of Science in Hubei, Peoples Republic of China. All samples were transported according to IATA international regulations for transporting viable samples.

### Serology Testing

An in-house anti-MERS-CoV IgG ELISA kit was developed based on the purified spike protein receptor binding domain. This highly sensitive and specific ELISA was previously validated for use with samples from camel and human (Zohaib *et al.*^2018^). This anti-MERS-CoV IgG ELISA was used with minor modifications. Camel samples were tested at 1:20 dilution and goat anti-camel IgG-horseradish peroxidase conjugate (Alpha Diagnostic International, San Antonio, TX, USA) was used as the secondary antibody at 1:3000. Based on the microneutralization test, a cut-off value of 0.35 was determined. For human samples, plasma was tested at a dilution of 1:20 and anti-human IgG-horseradish peroxidase conjugated monoclonal antibody (Kyab Biotech Co., Ltd, Wuhan, China) was used as the secondary antibody at 1:15000.

### Microneutralization Assay

A microneutralization assay was performed as described previously (Perera *et al*. 2013). Briefly, Vero B4 cells were seeded into 96-well plates. Plasma samples were incubated at 56 °C for 1 h. MERS-CoV (EMC strain) was diluted with DMEM to 100 TCID50/50 μL. The plasma samples were diluted by twofold in DMEM and incubated with MERS-CoV at 37 °C for 30 min. The medium was removed from the cells and 50 μL virus-plasma mixture was added. The virus-plasma mixture was removed after 1 h and 100 μL DMEM plus 2% fetal bovine serum (FBS) and 1% penicillin/streptomycin was added. The cells were incubated at 37 °C with 5% CO_2_, and the cytopathic effect (CPE) was observed and recorded at 4 days post-infection. Samples that inhibited CPE at a dilution of 1:20 were considered as positive.

### Molecular Detection of MERS-CoV in Camel Nasal Swabs

Viral RNA was extracted from camel nasal swabs stored in RNAlater^®^ (Ambion) using a viral RNA extraction kit (Roche, Basel, Switzerland) according to the manufacturer’s instructions. All camel nasal swabs were screened for MERS-CoV using two independent TaqMan quantitative reverse transcription PCR assays for the nucleocapsid gene (N) according to the WHO testing algorithm as described previously (Lu *et al.*^2014^). Additionally, camel samples positive according to RT-qPCR were also screened by MERS-CoV-specific RT-PCR targeting the *N* gene to confirm the presence of MERS-CoV in the samples as described previously (Corman *et al.*^2012^).

### MERS-CoV Isolation

Virus isolation from two positive nasal swabs with high viral loads was attempted using Vero cells. Vero cell monolayers were maintained in DMEM supplemented with 10% FBS. Nasal swab specimens in viral transport medium were diluted in DMEM before being added to the Vero cells. After incubation at 37 °C for 1.5 h, the inoculum was removed and replaced with fresh DMEM containing 2% FBS and antibiotics. The cells were incubated at 37 °C for 5 days and observed daily for CPEs. The culture supernatant and cells were examined for the presence of virus by the RT-qPCR N2 assay targeting the MERS-CoV *N* gene (Corman *et al*. 2012) and immunofluorescence assay using a rabbit antibody (prepared in house) against the MERS-CoV N protein.

### Full Genome Sequencing of MERS-CoV and Analysis

Libraries for next-generation sequencing were prepared using an Illumina Truseq *mRNA* kit (TruSeq Stranded mRNA Library Prep Kit, Cat #RS-122-2101, Illumina, San Diego, CA, USA) following the manufacturer’s instructions. The sequencing was performed on a HiSeq 3000 sequencer. The data obtained was analyzed in Metavisitor (a suite of galaxy tools) as described previously (Carissimo *et al*. 2017). The BLAST-guided scaffold was then used to reference align the reads in Geneious R11. Phylogenetic analysis was performed in MEGA7. For MERS-CoV samples that were not selected for full-genome sequencing, specific RT-PCRs were set-up to amplify the partial *S* gene and fragment covering the gene regions of *orf3*, *orf4a*, and *orf4b* as described previously (Smits *et al.*^2015^; Chu *et al.*^2018^).

### Statistical Analysis

ELISA-positive samples were statistically analyzed (Chi Square test) to detect associations between location, age, and sex. Univariable analysis was performed and odds ratios along with their 95% confidence intervals (CIs) were calculated. A *P* value < 0.05 was considered as significant in all analyses. Statistical analysis was performed in R (v3.5.1) with epicalc (v2.15.1.0) and the DescTools (v0.99.25) packages.

## Results

### MERS-CoV Seroprevalence among Camel Populations in Kenya

A total of 1163 plasma samples was collected from camels in 13 counties in Kenya between January 2016 to June 2018. Age and gender data for the 14 camels were missing. From the remaining 1149 samples, 801 (69.71%) were female and 348 (30.29%) were male. Most plasma samples (611; 52.54%) were collected from the northeastern part of Kenya (region C), which also has the largest herd of camels in Kenya and borders the Republic of Somalia. The demographic distribution of plasma in different camel breed regions and administrative counties is presented in Table 1 and Fig. 1. A total of 792 of the 1163 (68.10%) camel plasma samples tested positive by ELISA. Seroprevalence varied significantly (*P* < 0.001) among regions in the country, ranging from the highest in region B (79.86, 95% CI 74.90–84.06) followed by region C (75.29, 95% CI 71.72–78.54), region A (48.72, 95% CI 41.00–56.50), region E (42.11%, 95% CI 23.14–63.72), and region D (16.67%, 95% CI 10.20–26.05). The significantly (*P* < 0.001) highest prevalence was observed in Marsabit county (87.34%, 95% CI 78.24–92.98) (Supplemental Table S1). The seroprevalence of MERS-CoV increased with age and was significantly higher (*P* < 0.001) in adult camels > 7 years (82.37%, 95% CI 79.50–84.91) compared to sub-adults > 4 years < 7 years (58.57%, 95% CI 46.88–69.37) and juvenile camels < 4 years (36.05%, 95% CI 30.98–41.46). Significantly higher (*P* < 0.001) seroprevalence was observed in female (74.28%, 95% CI 71.14–77.19) than in male camels (53.74%, 95% CI 48.48–58.90). The geographical location and age of the camels were the main factors affecting the MERS-CoV seroprevalence in Kenya (Table 1).

### MERS-CoV Seroprevalence in Humans in Kenya

A total of 486 human plasma samples were collected from 10 counties in Kenya from March 2017 to June 2018, among which 231 (48.63%) subjects were male and 244 (51.37%) were female; gender data for 11 human samples were missing. Among the humans sampled, 95.27% stated that they have had close contact with livestock including camels. Three hundred people in this group stated they have regular contact with camels as herdsmen.

Twenty of the 486 human plasma samples showed positive results by ELISA. Of these, eight were from West Pokot, five were from Tana River, four were from Garissa, two were from Wajir, and one was from Isiolo Counties. Ten seropositive samples were male and nine were female, gender data of one ELISA reactive sample was missing. Twelve of the 20 ELISA-positive individuals were in frequent contact with camels. A micro-neutralization test was performed on all ELISA-reactive samples and none were positive in the neutralization assay.

### Molecular Detection of MERS-CoV

Eleven camel nasal swabs (Table 2) were positive for both N2 and N3 by RT-qPCR and nested PCR for the *N* gene. Among them, five were from adult camels, four were from juvenile camels, and one was a sub-adult camel. Age data for one positive camel was not available. High viral loads were observed in juvenile camels compared to in adults. Sequencing of the PCR-positive *N* gene samples revealed 100% identical sequences. Contamination was ruled out by repeating the experiments in independent laboratories and targeted PCR amplification and sequencing of the *N* gene. We partially sequenced the *S* gene of eight samples as described previously (Table 2) (Smits *et al.*^2015^). Of these eight, six samples were 100% identical, whereas the other two samples showed one nucleotide difference compared to the other six positive samples. All MERS-CoV isolates from Kenya clusters within sub-clade C2, which is associated with the African clade (data not shown).

**Table 2:** Detection of MERS-CoV genomes in eleven dromedary camels from Kenya.

Sample ID	Country	N2 assay	N3 assay	Nucleocapsid*	Spike*
C552 N	Marsabit	37.94	37.56	Positive	Negative
C290 N	Laikipia	38	38.15	Positive	Negative
C293 N	Tana River	37.87	36.88	Positive	Negative
C1214 N	Tana River	28.3	29.4	Positive	Positive
C1215 N	Tana River	23.8	24.2	Positive	Positive
C1272 N	Garissa	24.2	24.7	Positive	Positive
C1284	Garissa	30.6	32	Positive	Positive
C1295	Garissa	30.4	29.4	Negative	Positive
C1304	Garissa	26.8	26.3	Positive	Positive
C1308	Garissa	30.1	30	Positive	Positive
C1311	Garissa	27.6	26.7	Positive	Positive

*PCR using MERS-CoV-specific primers and subsequent confirmation by Sanger sequencing.

**Fig. 2:**
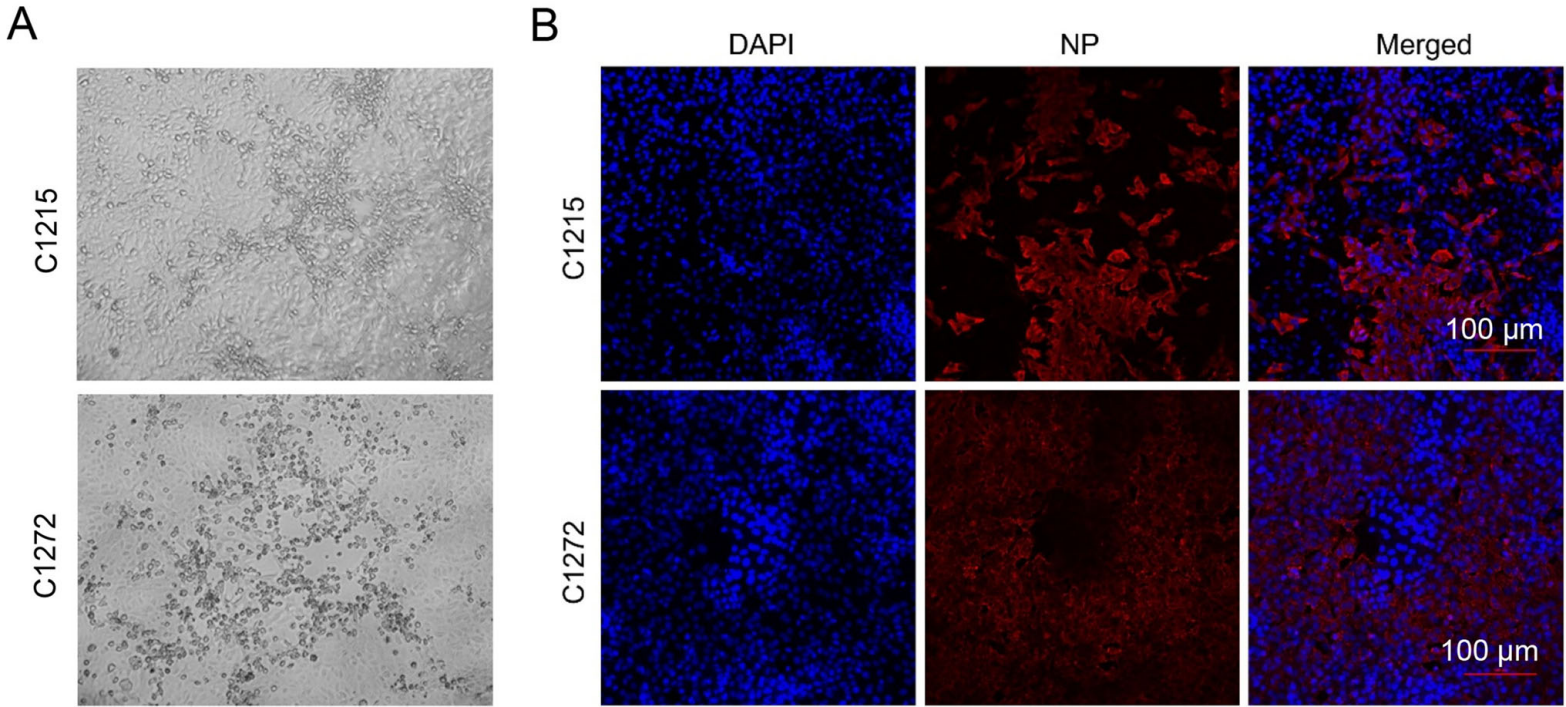
Isolation of camel MERS-CoV C1215 and C1272. **A** Induction of cytopathic effect on Vero cells. The images were taken by NIS Elements F (ECLIPSE TS100, Nikon). Original magnification: 100 ×. **B** Successful isolation of camel MERS-CoV was confirmed by immunofluorescent antibody staining using rabbit antibody against the MERS-CoV N protein. The columns (from left to right) show staining of nuclei (blue), virus replication (red), and both nuclei and virus replication (merged double-stain images). The images were taken by a confocal microscope. Scale bar = 100 μm.

**Fig. 3:**
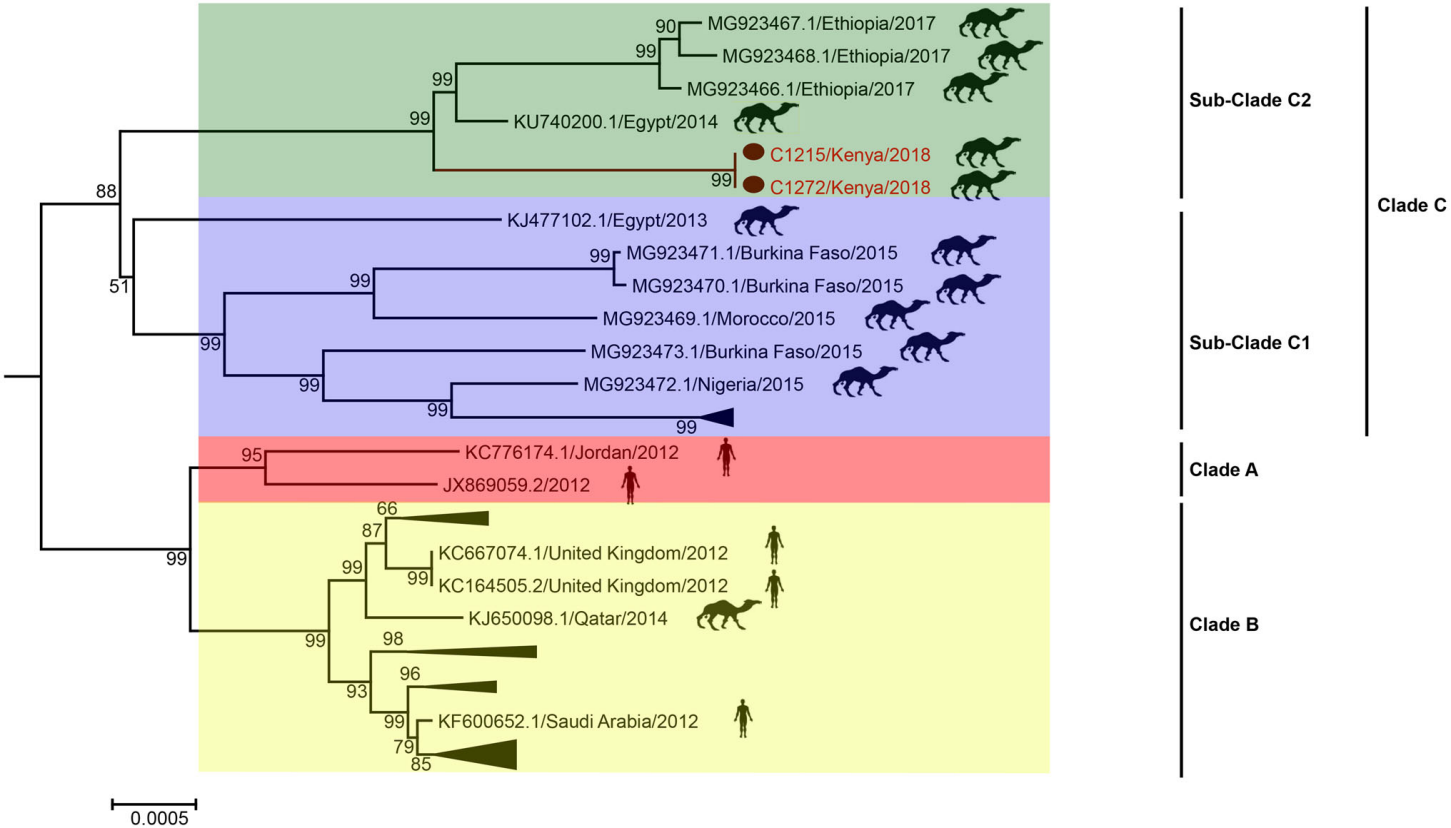
Phylogenetic analysis of MERS-CoV full genomes using neighbor-joining method in MEGA7. Bootstrap values of nodes are shown. Bootstrap values along branches are for 1,000 replicates. The tree was rooted against a MERS-CoV related bat coronavirus Neoromicia/PML-PHE1/RSA/2011 (KC869678) from South Africa. To allow for greater resolution of the viruses of interest, the long branch of KC869678 was removed. Detected MERS-CoV viruses in this study are red colored and identified with circle node markers (●). Scale bar indicates nucleotide substitutions per site.

**Fig. 4:**
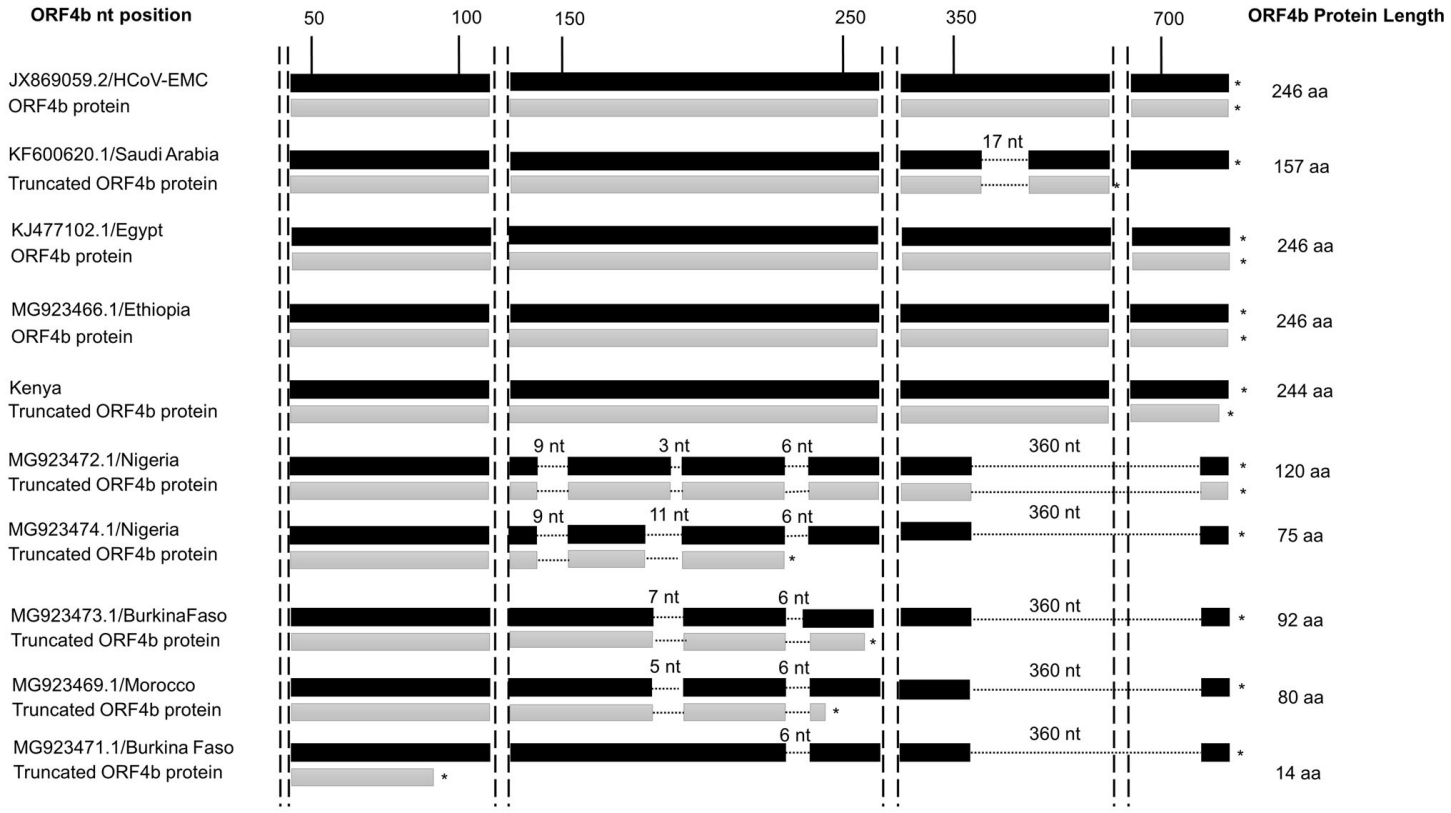
Schematic diagram showing the alignment of *orf4b* from Kenya and other previously reported MERS-CoV strains from Africa compared to HCoV-EMC. Putative ORFs are represented in black and proteins are shown as gray bars. Stop codon are indicated by asterisks and amino acid lengths are indicated.

Of the 11 samples detected in this study, two specimens with a high viral load were selected for virus isolation and full-length sequencing. Cytopathic changes in Vero cells were observed sequentially for 5 days post-infection (Fig. 2A). Camel MERS-CoV isolates in Vero cells were confirmed by immunofluorescence assay (Fig. 2B) and RT-qPCR (data not shown). Genetic nucleotide identity was 100% between two Kenyan viruses and 99.63%–99.77% for viruses from sub-clade C2 (Fig. 3), > 99.14% within camel and human MERS-CoV from the Middle East, and 97.82%–98.10% for viruses from sub-clade C1. Full-length sequencing was also performed on RNAlater^®^ preserved nasal swab samples. No nucleic acid differences were detected between isolated viruses and RNAlater^®^ preserved samples. The results of phylogenetic analysis of Kenyan MERS-CoV sequences along with other relevant viruses are shown in Fig. 3. The tree was rooted against a MERS-CoV-related bat coronavirus from South Africa (KC869678.4). Viruses from Kenya clustered together with sequences from Ethiopia, Egypt, Burkina Faso, Nigeria, and Morocco in clade C. Within clade C, viruses from Kenya clustered with viruses from Ethiopia and Egypt in sub-clade C2. Characteristics signature deletions in *orf4b* have been observed in viruses from Africa in sub-clade C1 but not in those from Egypt and sub-clade C2. Notably, Ethiopian and Egyptian MERS-CoV encode full-length *orf4b* (246 amino acids). Two viruses detected in this study were unique because they had a truncated *orf4b* (244aa) in sub-clade C2 (Fig. 4), whereas the other three sequences encoded full-length *orf4b* of 246 amino acids. Previously, a deletion pattern in the *orf3* region of African MERS-CoV has also been reported (Chu *et al.*^2018^). However, MERS-CoV from Kenya encodes full-length *orf3*. The amino acid residues of Kenyan sample C1215 differed from the EMC strain throughout the virus genome. The amino acid residues in the spike protein receptor binding domain of C1215 contained the amino acid substitutions S10F and S148P in the receptor binding motif. Although these two mutations were observed in the spike receptor binding domain region of MERS-CoV, the camel plasma and human plasma were effectively neutralized by the EMC strain from Kenya as described previously; thus these mutations likely do not affect the affinity of the protein for the host receptor (Corman *et al.*^2014^; Liljander *et al.*^2016^), although further studies are needed to confirm this.

## Discussion

Previous studies have reported the prevalence of MERS-CoV antibodies in archived samples (Corman *et al.*^2014^; Liljander *et al.*^2016^; Munyua *et al.*^2017^). This is the first study to report the detection and characterization of MERS-CoV from Kenyan dromedaries. The nucleic acid sequences determined in this study confirm the genetic uniqueness of the African clade of this virus compared to those reported in the Arabian Peninsula (Chu *et al.*^2018^). The observed genetic relationship between MERS-CoV sequences in Kenya and those detected in camels from Ethiopia are likely related to strong geographical affinities. A recent study characterized MERS-CoV from Africa, which exhibited region-dependent genetic diversity (Chu *et al.*^2018^). However, the study did not describe MERS-CoV from Kenya. In the current study, we found that MERS-CoV from Kenya is phylogenetically distinct (Clade C) from viruses in the Arabian Peninsula. Additionally, although truncation of *orf4b* was observed in Kenyan viruses, these viruses lacked the signature deletion patterns of *orf4b* observed previously in western African MERS-CoV in sub-clade C1. Previous studies focused on genetic and phenotypic characterization of MERS-CoV from West Africa; however, studies of Eastern African MERS-CoV utilizing one-health approaches are urgently needed.

This study further confirms the widespread presence of MERS-CoV among camels in all camel-rearing counties in Kenya. Although the seroprevalence varied between counties in Kenya, the widespread occurrence indicates the need for a national-wide strategy for early detection and prevention rather than county-based approaches. The seroprevalence rates observed in this study are consistent with those obtained in previous studies in Kenya and other African countries. Of the 13 counties evaluated in this study, the highest seropositivity was observed in Marsabit county (87.34%), which is comparable to that reported in a recent study of archived plasma of Marsabit county camels in 2013.

Eleven camels were positive for MERS-CoV nucleic acids in different counties, indicating the active circulation of MERS-CoV in Kenya, which was confirmed by the presence of MERS-CoV antibodies in juvenile/young camels in all geographic regions except for in region E. This agrees with the results of previous studies which suggested that younger camels are most likely to be infected by MERS-CoV (Sabir *et al.*^2016^). The presence of a high viral load in juvenile camels also suggests that younger camels play a key role in the maintenance and spread of MERS-CoV.

We were not able to sample an equal proportion of males and females mainly because of camel husbandry practices which value female camels, which produce milk, over males. Our study focused on high-risk groups i.e. humans that frequently interact with camels as well as infection hot spots such as common watering points and browsing locations. Most herders prefer moving camels in several groups for feeding and watering as a form of security. Additionally, watering points are frequented by different types of livestock such as cattle, sheep, and goats. Humans and wildlife also utilize these water sources, as these counties are in either arid or semi-arid regions where water is a scarce resource.

As previously reported, we were not able to detect neutralizing antibodies from sampled humans even though some of them were ELISA positive (Munyua *et al.*^2017^). We predict that MERS-CoV in Kenyan camels has low pathogenicity in humans, and neutralization antibody levels either decreased quickly or were undetectable in the neutralization assay; second, other coronaviruses are closely related to MERS-CoV but distinct at the virus neutralization epitopes. Additional studies focusing on surveillance in human, other wildlife species, and camels are needed to determine the prevalence of MERS-CoV in the general Kenyan population and identify the risk factors of infection.

## Electronic supplementary material

Below is the link to the electronic supplementary material.

Supplementary material 1 (PDF 61 kb)
