# Supplementary material for: Genetic Evidence of Middle East Respiratory Syndrome Coronavirus (MERS-Cov) and Widespread Seroprevalence among Camels in Kenya
Source: Virol Sin. 2018 Dec 20;33(6):484–92. doi: 10.1007/s12250-018-0076-4 (PMC6335226; doi:10.1007/s12250-018-0076-4)
Supplement: Supplementary file 1 — Supplementary material 1 (PDF 61 kb) [file 12250_2018_76_MOESM1_ESM.pdf]

VIROLOGICA SINICA

Electronic Supplementary Material

Genetic Evidence of Middle East Respiratory Syndrome Coronavirus (MERS-Cov) and Widespread Seroprevalence among Camels in Kenya

Sheila Ommeh[1#], Wei Zhang[2#], Ali Zohaib[2#], Jing Chen[2#], Huajun Zhang[2], Ben Hu[2], Xing-Yi Ge[3], Xing-Lou Yang[2], Moses Masika[4], Vincent Obanda[5], Yun Luo[2], Shan Li[2], Cecilia Waruhiu[2], Bei Li[2], Yan Zhu[2], Desterio Ouma[6], Vincent Odendo[6], Lin-Fa Wang[7], Danielle E. Anderson[7], Jacqueline Lichoti[8], Erick Mungube[6], Francis Gakuya[5], Peng Zhou[2], Kisa-Juma Ngeiywa[8,9], Bing Yan[2], Bernard Agwanda[10]✉, Zheng-Li Shi[2]✉

1. Institute for Biotechnology Research, Jomo Kenyatta University of Agriculture and Technology, Nairobi 62000-00200, Kenya
2. CAS Key Laboratory for Special Pathogens and Biosafety, Wuhan Institute of Virology, Chinese Academy of Sciences, Wuhan 430071, China
3. College of Biology, Hunan University, Changsha 410006, China
4. Department of Medical Microbiology, University of Nairobi, Nairobi 30197-00100, Kenya
5. Department, Kenya Wildlife Service, Nairobi 40241-00100, Kenya
6. Veterinary Research Institute, Kenya Agriculture and Livestock Research Organization, Nairobi 57811-00200, Kenya
7. Veterinary Services Programme in Emerging Infectious Diseases, Duke-NUS Medical School, Singapore 169857, Singapore
8. Directorate of Veterinary Services, State Department of Livestock, Ministry of Agriculture, Livestock Fisheries and Irrigation, Nairobi 34188-00100, Kenya
9. Kenya Camel Association, Nairobi 30095-00100, Kenya
10. Department of Zoology, National Museums of Kenya, Nairobi 40658-00100, Kenya

Supporting information to DOI: 10.1007/s12250-018-0076-4

Table S1. Detection of MERS-CoV from thirteen counties of Kenya, 2016-2018

Region	County	No. tested	No. positive	Prevalence% (95% CI)
Region A	West Pokot	67	19	28.36 (18.97–40.09)
	Turkana	74	51	68.92 (57.66–78.31)
	Baringo	15	6	40 (19.82–64.25)
Region B	Samburu	112	90	80.36 (72.05–86.66)
	Isiolo	102	75	73.53 (64.23–81.12)
	Marsabit	79	69	87.34 (78.24–92.98)
Region C	Mandera	93	66	70.97 (61.06–79.21)
	Wajir	103	82	79.61 (70.83–86.26)
	Garissa	198	145	73.23 (66.67–78.91)
	Tana River	217	167	76.96 (70.92–82.06)
Region D	Laikipia	84	14	16.67 (10.20–26.05)
Region E	Kitui	14	6	42.86 (21.38–67.41)
	Makueni	5	2	40 (11.76–76.93)

Table S2. Demographic characteristics of human samples tested in this study

Characteristics	Number (%)
Number of individuals	486
*Sex: Female number	244 (51.37)
Age:	
Median (range)	40 (1–100)
Mean (standard deviation)	41.77 (17.63)
Education level completed:	
No education	432 (89)
Preprimary/Primary	33 (7)
Secondary/Postsecondary	21 (4)

*Gender data of 11 samples were not available
